# Bioenergetic shift and actin cytoskeleton remodelling as acute vascular adaptive mechanisms to angiotensin II in murine retina and ophthalmic artery

**DOI:** 10.1016/j.redox.2020.101597

**Published:** 2020-05-29

**Authors:** Natarajan Perumal, Lars Straßburger, David P. Herzog, Marianne B. Müller, Norbert Pfeiffer, Franz H. Grus, Caroline Manicam

**Affiliations:** a Department of Ophthalmology, University Medical Centre of the Johannes Gutenberg University Mainz, Mainz, Germany; b Department of Psychiatry and Psychotherapy & Focus Program Translational Neurosciences (FTN), University Medical Centre of the Johannes Gutenberg University Mainz, Mainz, Germany

**Keywords:** Angiotensin II, Retina, Ophthalmic artery, Bioenergetics, Cytoskeleton, Glaucoma

## Abstract

Ocular vascular dysfunction is a major contributing factor to the pathogenesis of glaucoma. In recent years, there has been a renewed interest in the role of angiotensin II (Ang II) in mediating the disease progression. Despite its (patho)physiological importance, the molecular mechanisms underlying Ang II-mediated oxidative stress remain largely unexplored in the ocular vasculature. Here, we provide the first direct evidence of the alterations of proteome and signalling pathways underlying Ang II-elicited oxidative insult independent of arterial pressure changes in the ophthalmic artery (OA) and retina (R) employing an *in vitro* experimental model. Both R and OA were isolated from male C57Bl/6J mice (n = 15/group; n = 5/biological replicate) and incubated overnight in medium containing either vehicle or Ang II (0.1 μM) at physiological conditions. Label-free quantitative mass spectrometry (MS)-based proteomics analysis identified a differential expression of 107 and 34 proteins in the R and OA, respectively. Statistical and bioinformatics analyses revealed that protein clusters involved in actin cytoskeleton and integrin-linked kinase signalling were significantly activated in the OA. Conversely, a large majority of differentially expressed retinal proteins were involved in dysregulation of numerous energy-producing and metabolic signalling pathways, hinting to a possible shift in retinal cell bioenergetics. Particularly, Ang II-mediated downregulation of septin-7 (*Sept7*; *p* < 0.01) and superoxide dismutase [Cu-Zn] (*Sod1*; *p* < 0.05), and upregulation of troponin T, fast skeletal muscle (*Tnnt3*; *p* < 0.05) and tropomyosin alpha-3 chain (*Tpm3*; *p* < 0.01) in the OA, and significant decreased expressions of two crystallin proteins (*Cryab*; *p* < 0.05 and *Crybb2*; *p* < 0.0001) in the R were verified at the mRNA level, corroborating our proteomics findings. In summary, these results demonstrated that exogenous application of Ang II over an acute time period caused impairment of retinal bioenergetics and cellular demise, and actin cytoskeleton-mediated vascular remodelling in the OA.

## Highlights

• Acute Ang II stimulation elicits oxidative stress in ocular vasculature without pressor effect. .
• Dysregulation of energy-producing and metabolic pathways are implicated in the retina. .
• Actin cytoskeleton remodelling are vascular adaptation processes in the ophthalmic artery. .

## Graphical abstract

**Figure:**
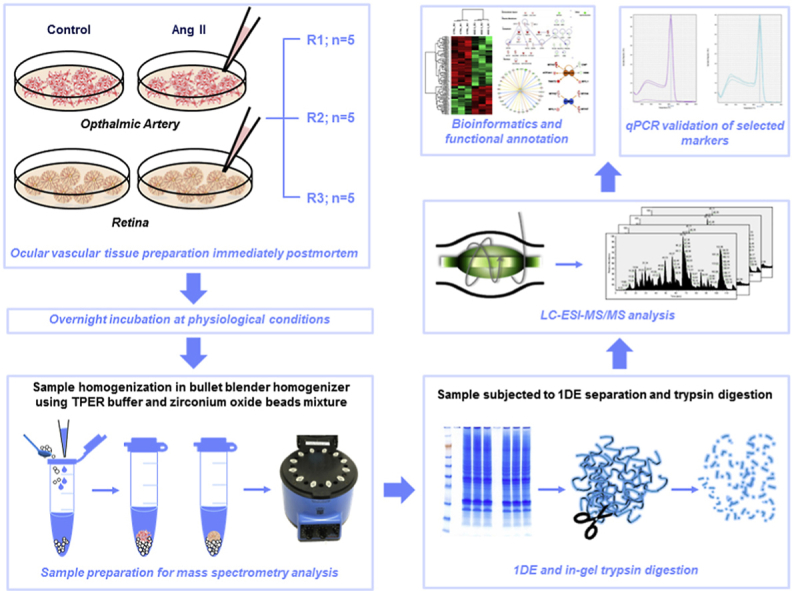

## Abbreviations

ACE: Angiotensin-converting enzyme
AMPK: Adenosine monophosphate-activated protein kinase
Ang II: Angiotensin II
CREB: cAMP-response element binding protein
EIF2: Eukaryotic translation initiation factor
FDR: False discovery rate
ILK: Integrin-linked kinase
qPCR: Quantitative real-time PCR
RAS: Renin-angiotensin system
Sept7: Septin-7
Sod1: Superoxide dismutase [Cu-Zn]
TCA cycle: Tricarboxylic acid cycle
Tnnt3: Troponin T, fast skeletal muscle
Tpm3: Tropomyosin alpha-3 chain

## 1 Introduction

The ocular system is an exceptionally metabolically active organ and despite its relatively small size, it ranks among the highest energy consumers, far exceeding the metabolic rate of the brain [1]. Therefore, it is undoubtable that the visual system is vulnerable to functional deficits induced by various antecedent factors including genetic predisposition, systemic diseases, oxidative stress, environmental conditions and external stimuli [[2], [3], [4], [5], [6]]. A salient feature in energy homeostasis involves the complex ocular vasculature. Blood vessels are highly dynamic components of the circulatory system that constantly undergo functional adaptation to various stimuli *via* vascular remodelling in order to supply blood to the different components of the eye with maximum efficiency. In recent years, it has been elegantly demonstrated that any imbalance and/or deficiencies in the vasculature of the visual system culminate in disease conditions [[7], [8], [9]]. This has been particularly conjectured in the pathogenesis of one of the most common yet deplorable ocular pathologies, glaucoma. Glaucoma is a term used to collectively define the heterogeneity of a multifaceted ocular disorder that causes glaucomatous optic neuropathy, which, if left unidentified and untreated, will ultimately result in irreversible visual loss [10,11]. One important factor in the pathogenesis of glaucoma is associated with impairment of blood flow as a consequence of vascular insufficiency in the eye [12,13].

While the vascular-related risk factor is not a new concept in glaucoma, there are several new aspects that warrant further investigation to unravel the underlying molecular mechanisms that govern the disease progression. Although considerable effort has been directed towards the study of intraocular blood vessels, particularly the retina, the functional relevance and importance of the retrobulbar vascular beds, which are the major blood suppliers to the optic nerve head, are often overlooked. Accumulating evidence has shown that the pathophysiology of progressive glaucoma is associated with circulatory anomalies in the major retrobulbar vessels comprising the ophthalmic and ciliary arteries [[14], [15], [16]].

One such system critically involved in the regulation of vascular tone and remodelling is the renin-angiotensin system (RAS) [17]. The activation of the primary effector molecule of RAS, angiotensin II (Ang II), leads to the generation of reactive oxygen species (ROS), which causes endothelial dysfunction and eventually, elicits deleterious effects in the vascular system, leading to vascular remodelling. In recent years, there has been a renewed interest in the role of Ang II in mediating the pathogenesis of glaucoma and angiotensin-converting enzyme (ACE) inhibitors have emerged as a new class of drugs for the treatment of glaucoma [17]. Despite its (patho)physiological importance, it has to be underscored that the mechanisms accounting for ocular vascular remodelling are as yet unexplored and there is still significant opportunity for exploration in this area.

In light of these factors, our present study investigated the direct effect of Ang II on the global proteome of two crucial ocular vascular beds consisting of the ophthalmic artery, which represents a retrobulbar blood vessel and the intraocular retinal tissue. We tested the hypothesis that the retina is more susceptible to Ang II-elicited oxidative insult than the ophthalmic artery in an *in vitro* experimental mouse model. The schematic overview of the experimental design employed to execute this study is depicted in the graphical abstract.

## 2 Materials and methods

### 2.1 Animals

All experiments using animals were conducted in strict adherence to the Association for Research in Vision and Ophthalmology (ARVO) Statement for the Use of Animals in Ophthalmic and Vision Research and, animal care conformed to the institutional guidelines and the EU Directive 2010/63/EU for animal experiments. This study was approved by the institutional animal care committee [Translational Animal Research Centre (TARC)] of the University Medical Centre of the Johannes-Gutenberg University Mainz. Animal use in this study was in accordance with the 3R principle. Male C57BL/6J mice (The Jackson Laboratory, Bar Harbour, ME, USA) aged 3 to 5 months old were used in this study. Animals were housed under standardised conditions (12 h light/dark cycle, temperature 23 ± 2 °C and humidity 55 ± 10%) and were provided standard mouse chow and water *ad libitum*.

### 2.2 Experimental protocol

Mice were sacrificed under CO_2_ followed by immediate enucleation of their eyes with attached optic nerve and extraocular tissues in ice-cold Krebs-Henseleit buffer at pH 7.4 with the following ionic composition in mM: 118.3 NaCl, 4.7 KCl, 2.5 CaCl_2_, 1.2 MgSO_4_, 1.2 KH_2_PO_4_, 25 NaHCO_3_, and 11 glucose (Carl Roth GmbH, Karlsruhe, Germany). Next, both ophthalmic artery and retina were isolated under a stereomicroscope using precision tweezers with fine tips and a pair of straight Vannas capsulotomy scissors. Care was taken to not stretch or damage the blood vessels and retinal tissues during isolation. The method for *in vitro* vessel incubation in Ang II is according to Didion *et al*. [18] with modifications. Briefly, isolated vessels and retinae were incubated in individual wells using 6-well cell culture plates containing DMEM without phenol red, 120 U/mL penicillin and 10 mg/ml streptomycin with either vehicle (deionized water) or Ang II (0.1 μM) (Sigma-Aldrich, Germany) for 22 h at physiological conditions. Samples were subjected to protein extraction steps after incubation.

### 2.3 Tissue protein extraction

The protein extraction procedure was carried out according to our established method catered specifically for small vascular beds, as described in detail elsewhere [19,20]. Briefly, the ophthalmic artery and retinal tissue were subjected to homogenization in a bullet blender homogenizer (BBY24M Bullet Blender Storm, Next Advance Inc., Averill Park, NY, USA) using T-PER Tissue Protein Extraction Reagent (Thermo Scientific Inc., Waltham, MA, USA). The supernatant collected following centrifugation at 10 000*g* for 5 min was subjected to buffer exchange and cleaning step using 3 kDa centrifugal cut-off filters (Amicon Ultra 0.5 mL, Merck Millipore, Carrigtwohill, Ireland). Protein concentration was determined employing the bicinchoninic acid (BCA) protein assay kit (Pierce, Rockford, IL, USA).

### 2.4 One-dimensional gel electrophoresis (1DE)

Triplicates of samples in each group were subjected to 1DE (50 μg/lane) and separated under reducing conditions on 10-well precast 4–12% Bis-Tris mini gels with 1x NuPAGE MES SDS Running Buffer (both from Thermo Fisher Scientific, Rockford, IL, USA). Gels were run for 60 min at 4 °C at a constant voltage of 150 V. Pre-stained protein standard, SeeBlue Plus 2 (Thermo Fisher Scientific, Rockford, IL, USA) was used as molecular mass marker and Novex Colloidal Blue Staining Kit (Invitrogen, Karlsruhe, Germany) was used to stain the gels according to the manufacturer's instructions. Subsequently, gels were destained overnight to eliminate background staining and scanned on Epson Perfection V600 Photo Scanner (Seiko Epson Corporation, Suwa, Nagano, Japan). Protein bands in each lane were excised (each gel lane were sliced into 20 pieces), reduced, alkylated and tryptic-digested, as described previously [19,20]. In-gel tryptic-digested peptides were further purified using ZipTip C18 pipette tips (Millipore, Billerica, MA, USA), concentrated to dryness in a vacuum centrifuge and reconstituted with 0.1% trifluoroacetic acid (TFA) prior to LC-MS/MS analysis.

### 2.5 Liquid chromatography-electrospray ionization-MS/MS (LC-ESI-MS/MS) analysis

LC-MS/MS measurements were performed using the Hybrid Linear Ion Trap-Orbitrap MS system (LTQ Orbitrap XL; Thermo Scientific, Bremen, Germany), as described in detail elsewhere [19,20]. The reverse phase aqueous solvent A consisted of LC-MS grade water with 0.1% (v/v) formic acid and the organic solvent B consisted of LC-MS grade acetonitrile with 0.1% (v/v) formic acid. The gradient had a running time of 60 min per gel band, as follows: 0–35 min: 15–40% B, 35–40 min: 40–60% B, 40–45 min: 60–90% B, 45–50 min: 90% B, 50–53 min: 90-10% B and 53–60 min: 10% B. The general parameters of the instrument are as described in our previous studies [19,20]. Tandem data was obtained by selecting top five most intense precursor ions provided by the high-resolution MS scan of the Orbitrap-Fourier transform mass spectrometry (FTMS) analyser and subjected them for further fragmentation by collision-induced dissociation (CID) employing the normalized collision energy (NCE) of 35% with activation time of 30 ms, repeat count of 3 and dynamic exclusion duration of 600 s. The resulting fragmented ions were recorded in the LTQ.

### 2.6 Label-free quantification (LFQ)

The acquired continuum MS spectra were analysed employing the MaxQuant computational proteomics platform version 1.6.1.0 (http://www.maxquant.org) with a built-in Andromeda search engine for peptide and protein identification and, LFQ and intensity-based absolute quantification (iBAQ) algorithm enabled [20,21]. The tandem MS spectra were searched against UniProt databases for *Mus musculus* and *Homo sapiens* with the following standard settings: Peptide mass tolerance of ±30 ppm, fragment mass tolerance set to ±0.5 Da with ≥6 amino acid residues and only ‘unique plus razor peptides’ that belong to a protein were chosen, trypsin as enzyme and maximum number of missed cleavages sites set to 2, carbamidomethylation of cysteine as fixed modification and oxidation of methionine and acetylation of N-termini as variable modification. Database search for peptide identification was conducted using two different databases owing to the limited availability of mouse proteins and also in order to maximize peptide identification, which was instrumental for subsequent detailed functional characterization [[22], [23], [24], [25]]. A target-decoy based false discovery rate (FDR) of <1% was used for peptide and protein identification. The summary of MaxQuant parameters employed in the current analyses for both ophthalmic artery and retina is tabulated in **Supplementary Data 4 and 5**, respectively.

### 2.7 Statistical and bioinformatics analysis of proteomics data

The MaxQuant-generated output data table “proteingroups.txt” was filtered for contaminants and reverse hits prior to statistical analysis with Perseus software (version1.6.2.3). First, Pearson's correlation coefficients were analysed employing the normalized LFQ intensity dataset to assess experimental reproducibility and the homogeneity of the designated groups. Next, LFQ intensities of all identified proteins were log_2_-transformed and missing values were imputated with values generated from a normal distribution (width: 0.3 and down shift: 1.8). Significantly differentially expressed proteins were identified by a Student's two-sample *t*-test with p < 0.05. Unsupervised hierarchical clustering analysis was performed according to Euclidean distance (linkage = average; preprocess with k-means) to illustrate the heat map of the differentially expressed proteins. The gene names of these significantly differentially expressed proteins in each group were used for subsequent functional annotation and pathways analyses employing Ingenuity Pathway Analysis software (v01–04, IPA; Ingenuity QIAGEN Redwood City, CA) (https://www.qiagenbioinformatics.com/products/ingenuity-pathway-analysis) [26]. Further details of the IPA analysis can be found in our previous publications [19,21].

### 2.8 RNA extraction and cDNA synthesis

Total RNA was extracted from ophthalmic arterial and retinal samples by a combination of the RNeasy Micro Kit (Qiagen, Germany) in combination with TRIzol® (ThermoFisher, Germany). Disruption and homogenization of the tissue was performed as follows: samples were flash-frozen in liquid nitrogen, lysed and homogenized in 800 μl ice-cold TRIzol® using a potter and syringes. Samples were vortexed for 30 s and incubated for 5 min at room temperature. For the phase separation, Phase Lock Gel-Heavy tubes (PLG, Thermo Fisher Scientific) were used. Chloroform (160 μl) was added to the PLG tube, shaken vigorously for 15 s and incubated for 3 min at room temperature. Phase separation was achieved by centrifugation at 12 000*g* for 15 min at 4 °C. The clear, aqueous top phase containing the RNA was transferred to a fresh tube. Subsequent RNA extraction was performed according to the manufacturer's protocol (RNeasy Micro Kit, Qiagen, Germany) and freshly diluted for application. Quality control of RNA samples was performed using Qubit® (ThermoFisher, Germany) concentration measurement and Bioanalyser profiling (Agilent Technologies, Germany). Concentrations were >65 ng/μl, with RIN values > 6.2 for the ophthalmic arterial samples. Concentrations were >400 ng/μl, with RIN values > 7.9 for retinal samples. 500 ng of RNA was used to synthesize cDNA according to the manufacturer's protocol with the PrimeScript™ RT Master Mix (Takara Bio Inc., Japan).

### 2.9 Quantitative real-time PCR (qPCR)

We performed qPCR using the SYBR® Green PCR Master Mix (ThermoFisher, Germany) and a StepOnePlus qPCR device (ThermoFisher, Germany). Primer pairs were customized using primer-BLAST (https://www.ncbi.nlm.nih.gov/tools/primer-blast/) and ordered from Sigma Aldrich (Germany). Primer specificity was tested with melting curves and agarose gels before use. PCR cycling conditions were as follows: 15 min at 95 °C; [15 s at 94 °C, 30 s at 55 °C, 30 s at 72 °C] x 40 cycles. Two housekeeping genes (HPRT1 and GAPDH) were used to normalize mRNA of genes of interest using the ΔΔct method. Data was normalized and compared to control values set at 1.0.

### 2.10 Statistical analysis of qPCR

Sample sizes are indicated in the figure legends. The values are displayed as mean ± SEM. Data was plotted and descriptive statistics were applied to check for normal distribution of the data (D'Agostino & Pearson omnibus normality test). Normally distributed data was analysed using the unpaired two-tailed T-test. Alpha was set at 5%, with p values < 0.05 considered statistically significant. Data was analysed using Prism 5 software (GraphPad, USA).

## 3 Results

### 3.1 Label free quantitative proteomics analysis

**Fig. 1:**
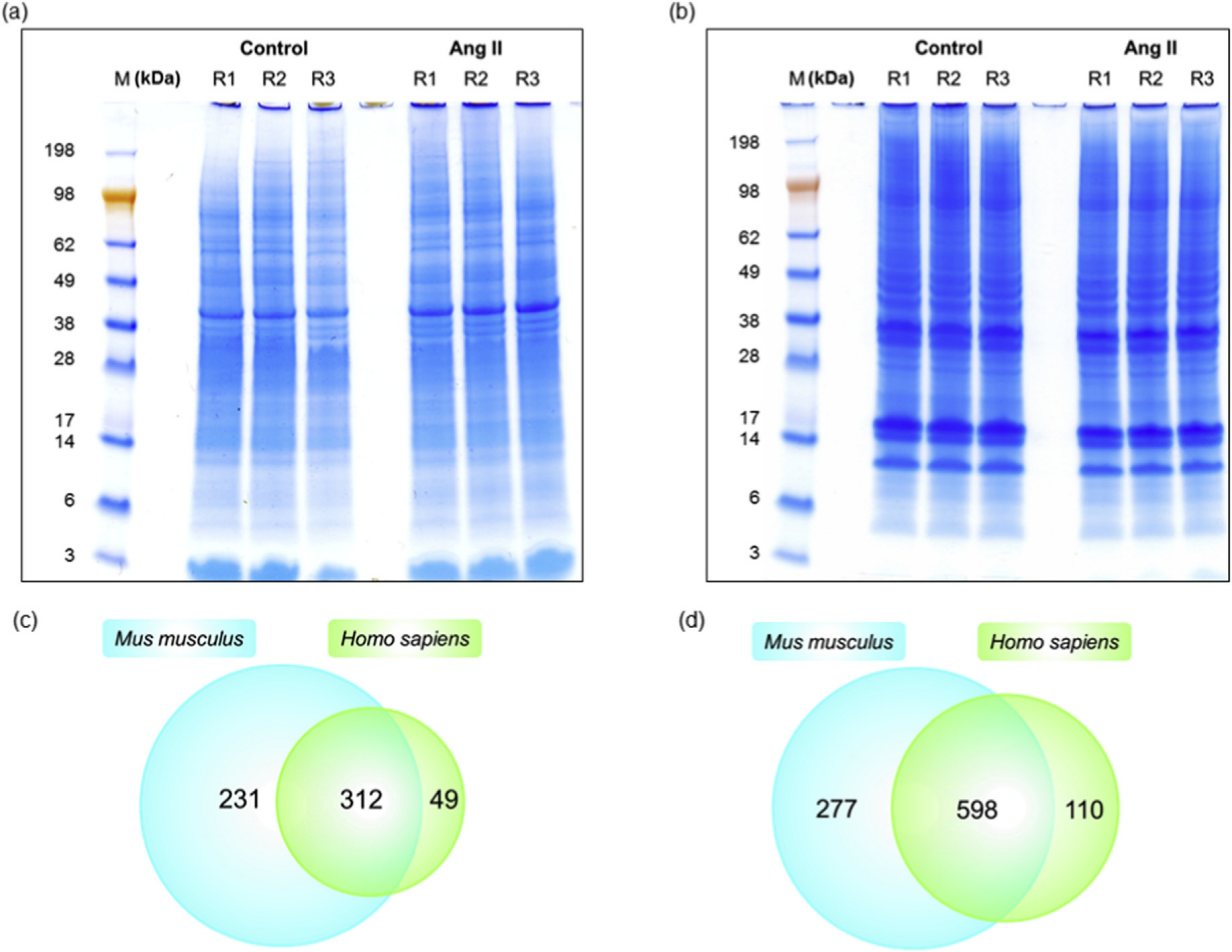
**Proteome of ophthalmic artery and retina.** (a) Representative protein profiles of (a) ophthalmic artery and (b) retina subjected to Ang II and vehicle resolved in 1DE gel and stained with Colloidal blue. Venn diagrams depict the total number of proteins identified in both mouse and human databases in the (c) ophthalmic artery and (d) retina. M: Marker; R1-R3 represent three biological replicates. (For interpretation of the references to colour in this figure legend, the reader is referred to the Web version of this article.)

Protein profiles of the ophthalmic artery and retina subjected to Ang II in comparison to their respective controls resolved in 1DE are as shown in Fig. 1a and b, respectively. Combined bottom-up LC-MS/MS proteomics analysis of triplicates of both groups identified a total of 592 and 985 proteins from the ophthalmic artery and retina, respectively, at less than 1% (<1%) false discovery rate (FDR) (complete data in **Supplementary Data 1**). In this study, both mouse and human UniProt databases were used for tandem MS search to maximize protein identification because of limited annotation of *Mus musculus* proteins (16 991 proteins) compared to the *Homo sapiens* database (20 394 proteins). Moreover, it has been highlighted that using multiple database search can provide significantly more detailed information, which are not restricted to the sequences obtained from only the organism of interest [[22], [23], [24], [25]]. The number of proteins identified in both databases for both samples are illustrated in the Venn diagrams in Fig. 1c and d. A large majority of identified proteins were found to be expressed in both mouse and human databases, with 52.7% (312 proteins) and 60.7% (598 proteins) in the ophthalmic artery and retina, respectively. A higher percentage of the proteins were exclusively identified from the mouse database, with 39% (231 proteins) and 28.1% (277 proteins) in the ophthalmic artery and retina, respectively; while lesser number of proteins were identified from the human database for both samples with 8.3% (49 proteins) and 11.2% (110 proteins) in the ophthalmic artery and retina, respectively (complete data in **Supplementary Data 2**).

### 3.2 The effect of Ang II on ophthalmic arterial proteome

**Fig. 2:**
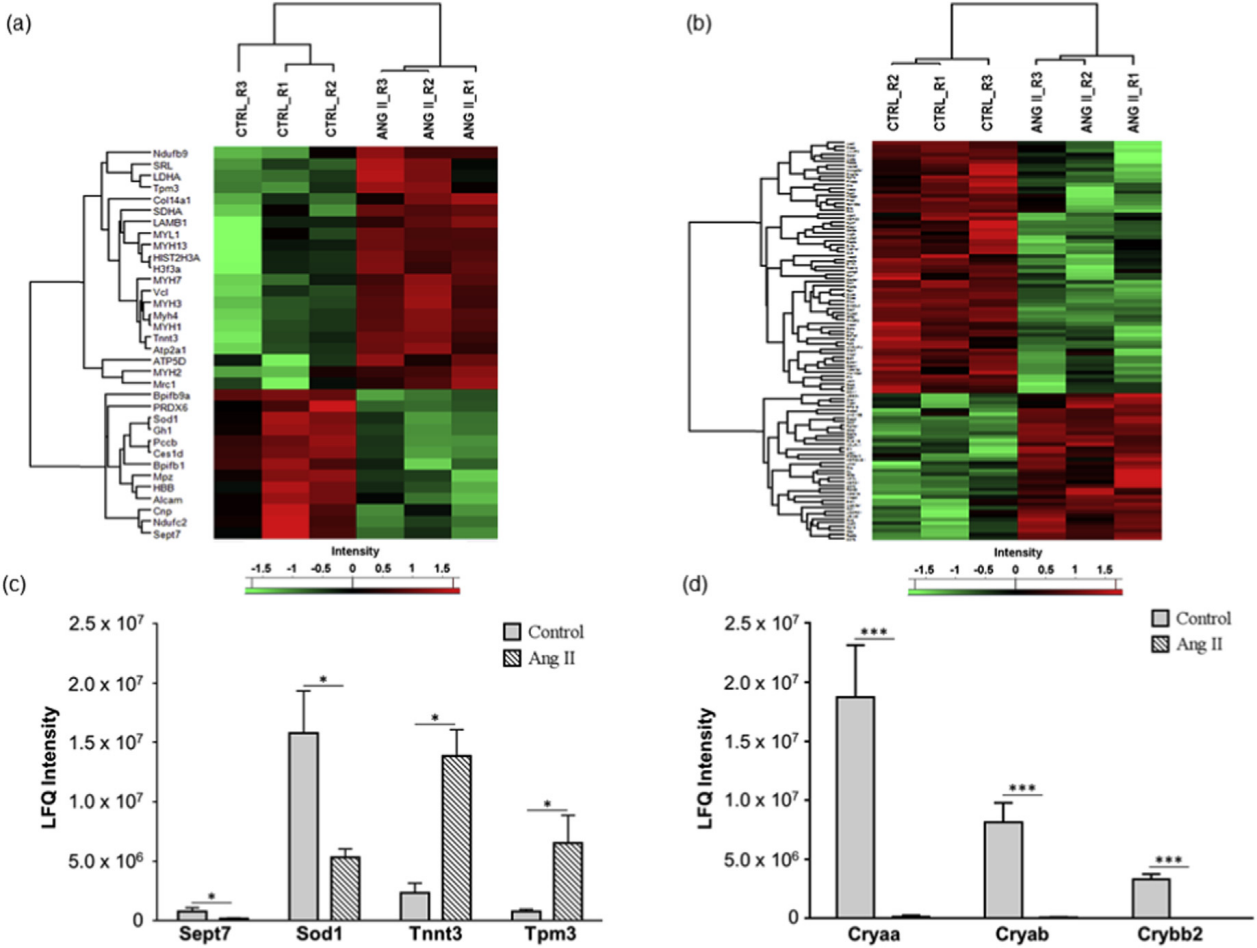
**Differential expression profiles of ophthalmic arterial and retinal proteins**. The hierarchical clustering of the differentially expressed proteins represented in a heat map for (a) ophthalmic artery and (b) retina. The upregulated proteins are shown in red and the downregulated proteins are in green. R1-R3: Biological replicate 1 to 3. Bar charts showing the expression profiles of highly significant exemplary proteins in the Ang II group compared to control in the (c) ophthalmic artery and (d) retina. The expressions of *Sept7* and *Sod1* were significantly decreased, while *Tnnt3* and *Tpm3* were increased in the ophthalmic artery. All three crystallin proteins were significantly decreased or absent in the retinal samples subjected to Ang II. *p < 0.05; ***p < 0.001. (For interpretation of the references to colour in this figure legend, the reader is referred to the Web version of this article.)

**Fig. 3:**
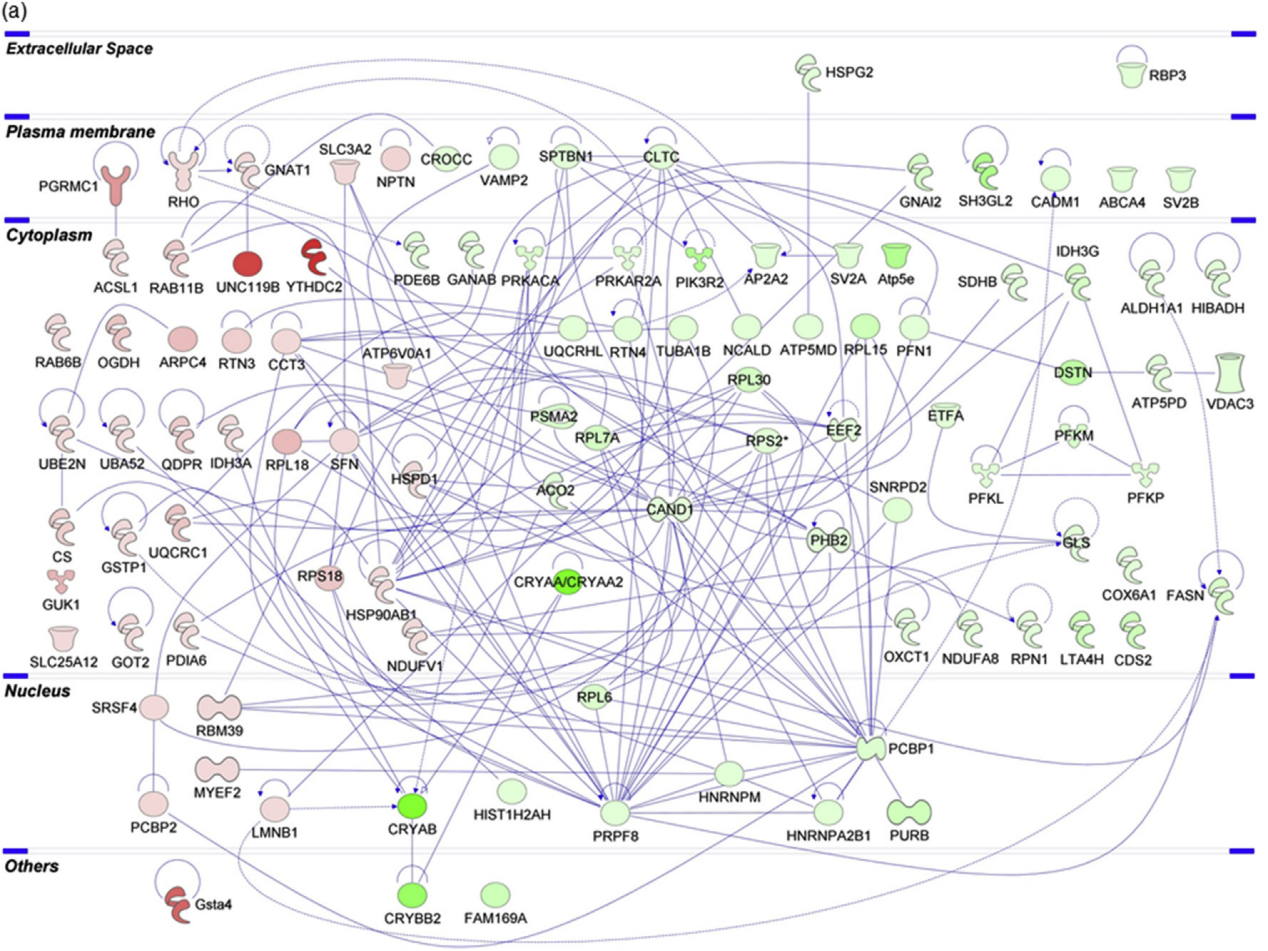

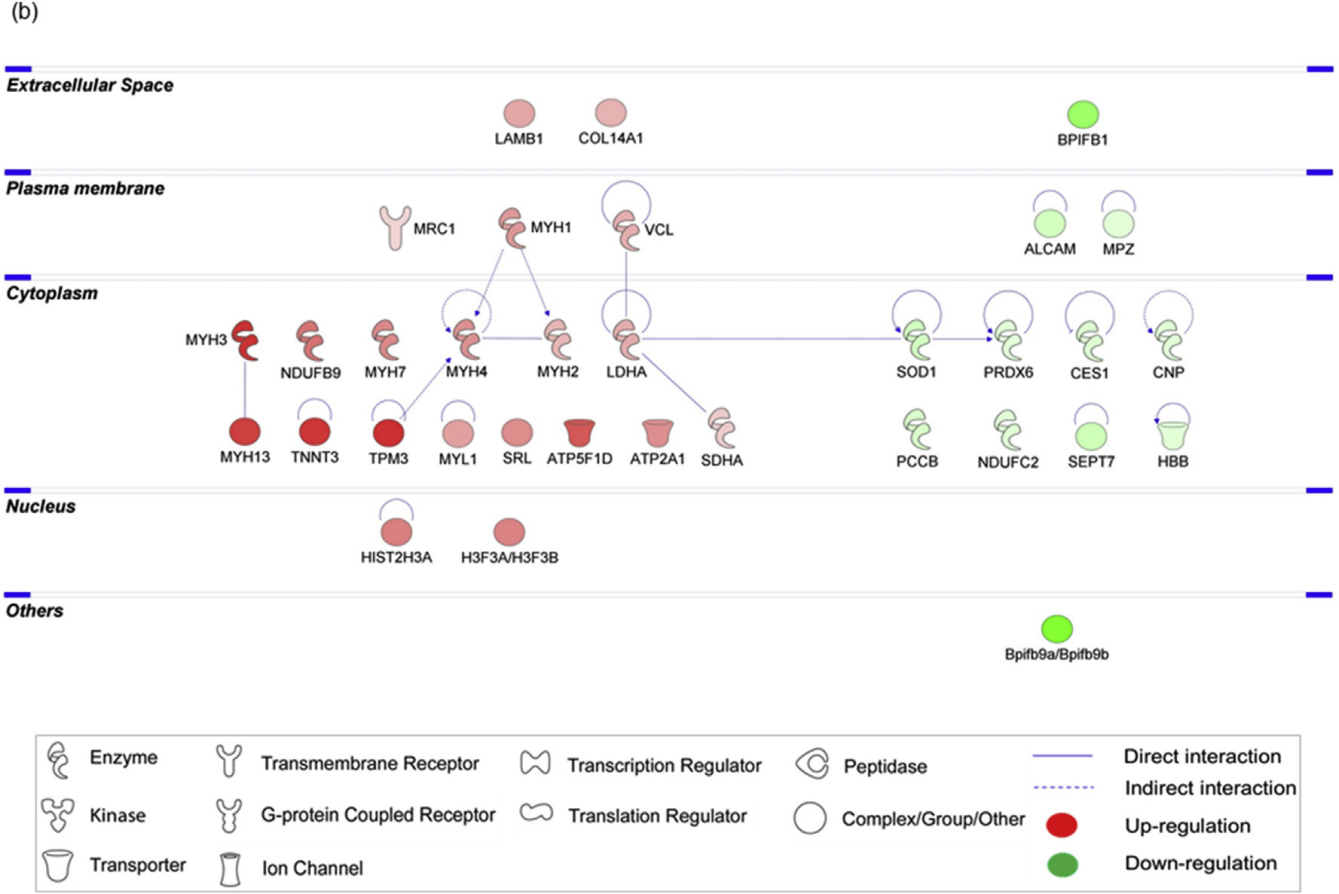
**Protein-protein interaction (PPI) networks of the differentially expressed proteins of ocular vascular beds**. The PPI generated by IPA analysis depicts the networks of differentially expressed proteins in the (a) ophthalmic artery and (b) retina. Colours red and green represent increment and decrement of protein abundance, respectively; with different colour intensities that correspond to the degree of differential expression. Proteins are annotated according to their cellular localization and are depicted as different shapes, which represent the functional classes of the proteins (e.g. enzyme, transporter, ion channel, etc.). (For interpretation of the references to colour in this figure legend, the reader is referred to the Web version of this article.)

**Fig. 4:**
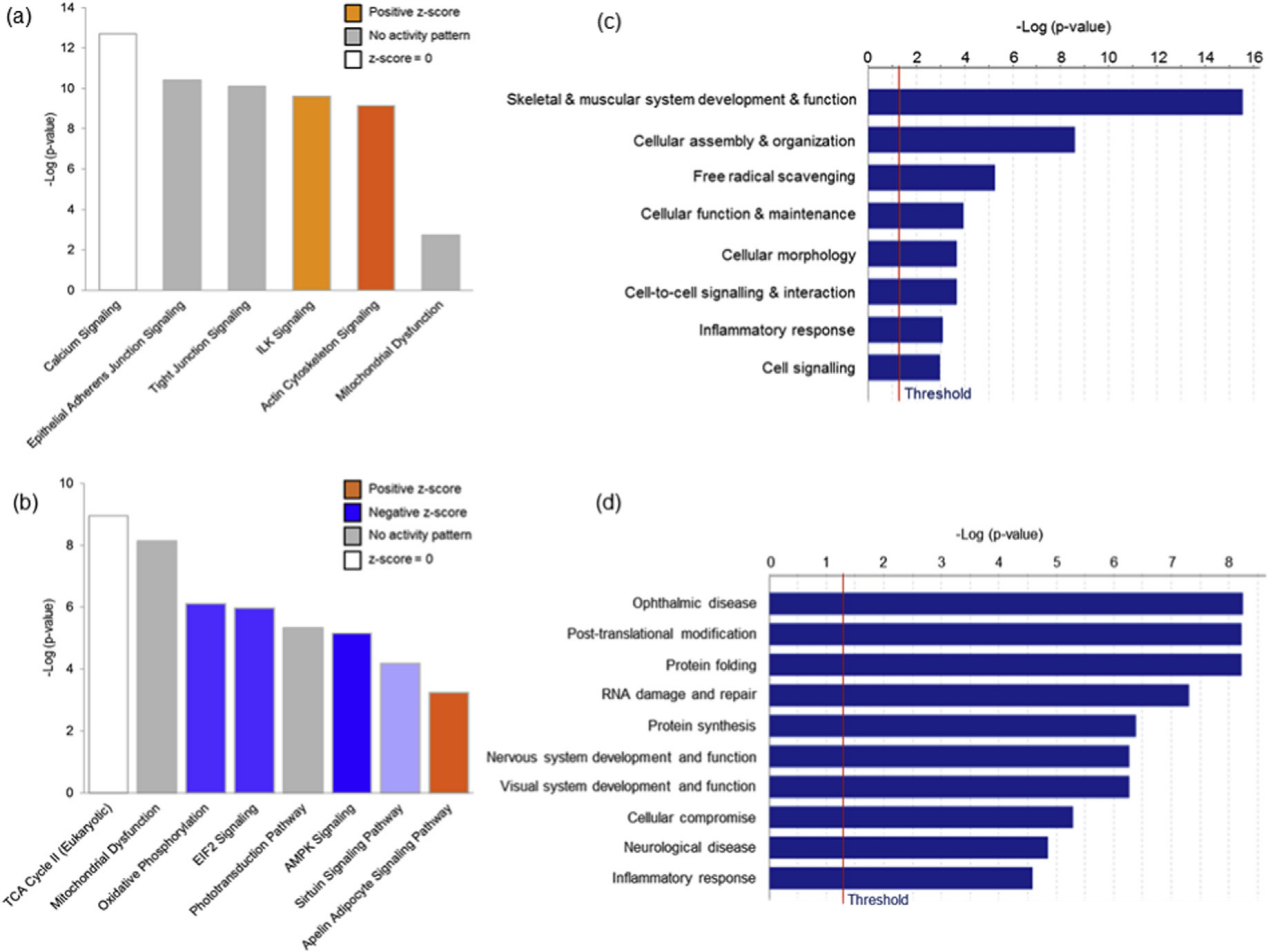
**Top significantly affected canonical pathways and diseases and biological functions**. Bar charts depict the significantly (*p* < 0.001) enriched canonical pathways determined by *p*-value overlap between the proteins identified in our datasets and the molecules in the respective pathways, implicated in the (a) ophthalmic artery and (b) retina following Ang II treatment. Overall z-scores are represented by the colour orange, which indicates activation and blue indicates inhibition of the signalling pathways; grey indicates indeterminable activity pattern. Negative log_10_-transformed *p*-values of the top significant diseases and biological functions of the differentially expressed proteins are represented by horizontal bar charts for (c) ophthalmic artery and (d) retina. (For interpretation of the references to colour in this figure legend, the reader is referred to the Web version of this article.)

The Ang II-mediated vascular proteome changes were demonstrated by the differential expression of 34 proteins, with 21 up- and 13 down-regulated proteins, as tabulated in Table 1 and depicted in heat map in Fig. 2a. Exemplary regulation profiles of several highly differentially expressed proteins in the two groups are shown in Fig. 2c, where Sept7 and Sod1 were significantly down-regulated (*p* < 0.05), while Tnnt3 and Tpm3 were significantly up-regulated (*p* < 0.05). The IPA analysis of both clusters revealed that the large majority of these differentially expressed proteins comprise enzymes, which are localized in the cytoplasm (Fig. 3b). Further analysis demonstrated that the top significantly affected canonical pathways were involved mainly in calcium signalling (*p* = 2.09E-13), epithelial adherens junction (*p* = 3.93E-11) and tight junction signalling (*p* = 8.31E-11), integrin-linked kinase (ILK) signalling (*p* = 5.07E-12), actin cytoskeleton signalling (*p* = 7.08E-10) and implicated in mitochondrial dysfunction (*p* = 1.91E-03) (Fig. 4a). Moreover, the disease and biological functional analysis of the differentially expressed proteins showed that most protein clusters were significantly associated with skeletal and muscular system development and function (*p* = 2.74E-16), cellular functional maintenance mechanisms such as cell assembly and organization (*p* = 2.52E-09), cellular morphology (*p* = 2.10E-04), cell-cell signalling (*p* = 2.10E-04) and also in free radical scavenging (*p* = 5.42E-06) and inflammatory responses (*p* = 8.00E-04) (Fig. 4c).

**Table 1:** List of differentially expressed ophthalmic arterial proteins following acute treatment to Ang II.

Protein ID	Protein name	Gene name	*p*-value	Log_2_ fold-change
Up-regulated proteins				
P21107	Tropomyosin alpha-3 chain	Tpm3	2.26E-02	2.9203
P11055	Myosin-3	MYH3	5.24E-03	2.85156
Q9QZ47	Troponin T, fast skeletal muscle	Tnnt3	1.45E-02	2.73424
Q9UKX3	Myosin-13	MYH13	4.31E-02	2.57876
P30049	ATP synthase subunit delta, mitochondrial	ATP5D	3.68E-02	2.18681
Q9CQJ8	NADH dehydrogenase [ubiquinone] 1 beta subcomplex subunit 9	Ndufb9	2.65E-02	1.77138
Q71DI3	Histone H3.2	HIST2H3A	2.37E-02	1.58786
P84244	Histone H3.3	H3f3a	2.54E-02	1.54917
P12883	Myosin-7	MYH7	1.09E-02	1.46828
Q86TD4	Sarcalumenin	SRL	3.05E-02	1.40611
Q8R429	Sarcoplasmic/endoplasmic reticulum calcium ATPase 1	Atp2a1	1.15E-02	1.3743
Q5SX39	Myosin-4	Myh4	5.36E-03	1.37425
P12882	Myosin-1	MYH1	7.46E-03	1.24815
P05976	Myosin light chain 1/3, skeletal muscle isoform	MYL1	3.88E-02	1.14966
P07942	Laminin subunit beta-1	LAMB1	3.61E-02	1.06085
P00338	l-lactate dehydrogenase A chain	LDHA	4.07E-02	1.00148
Q64727	Vinculin	Vcl	1.12E-02	0.971758
Q80X19	Collagen alpha-1(XIV) chain	Col14a1	1.70E-02	0.918142
Q9UKX2	Myosin-2	MYH2	4.97E-02	0.889952
P31040	Succinate dehydrogenase [ubiquinone] flavoprotein subunit, mitochondrial	SDHA	1.82E-02	0.615652
Q61830	Macrophage mannose receptor 1	Mrc1	3.95E-02	0.51224
Down-regulated proteins				
Q80XI7	Vomeromodulin	Bpifb9a	3.70E-04	-6.44219
Q61114	BPI fold-containing family B member 1	Bpifb1	1.34E-02	-4.05153
P06880	Somatotropin	Gh1	1.47E-02	-3.91578
O55131	Septin-7	Sept7	4.83E-02	-1.72606
Q61490	CD166 antigen	Alcam	4.48E-02	-1.54606
P08228	Superoxide dismutase [Cu-Zn]	Sod1	2.38E-02	-1.51161
Q99MN9	Propionyl-CoA carboxylase beta chain, mitochondrial	Pccb	4.62E-03	-1.25907
Q8VCT4	Carboxylesterase 1D	Ces1d	3.15E-03	-1.07386
Q9CQ54	NADH dehydrogenase [ubiquinone] 1 subunit C2	Ndufc2	2.72E-02	-1.03234
P68871	Hemoglobin subunit beta	HBB	4.76E-02	-1.0026
P16330	2,3-cyclic-nucleotide 3-phosphodiesterase	Cnp	4.66E-02	-0.941758
P27573	Myelin protein P0	Mpz	2.49E-02	-0.630318
P30041	Peroxiredoxin-6	PRDX6	3.90E-02	-0.371027

### 3.3 The effect of Ang II on retinal proteome

A total of 107 proteins were found to be differentially expressed in the retina samples incubated with Ang II compared to control (full list in **Supplementary Data 3**). Among these, a higher number of proteins (68 proteins) were down-regulated, while 39 proteins were up-regulated, as represented in the heat map (Fig. 2b). Particularly, the regulation of a cluster of crystallin proteins was found to be significantly down-regulated (*p* < 0.001), which is exemplified in Fig. 2d. Similar to the ophthalmic arterial proteins, the protein-protein interaction networks of the differentially expressed retinal proteins demonstrate that a high number of proteins were localized in the cytoplasm and composed of enzymes (Fig. 3a). The top canonical pathways ranked by *p*-value are depicted in Fig. 4b, with an overall trend of decreased activity, as evidenced by the negative z-scores of the four major pathways comprising the oxidative phosphorylation (z-score = -1.134), eukaryotic translation initiation factor (EIF2) signalling (z-score = -1.134), adenosine monophosphate-activated protein kinase (AMPK) signalling (z-score = -1.414) and the sirtuin signalling pathways (z-score = -0.378). Strikingly, all proteins implicated in the AMPK signalling, composed of elongation factor 2 (Eef2), fatty acid synthase (Fasn), ATP-dependent 6-phosphofructokinase, liver type (Pfkl), ATP-dependent 6-phosphofructokinase, muscle type (Pfkm), ATP-dependent 6-phosphofructokinase, platelet type (Pfkp), phosphatidylinositol 3-kinase regulatory subunit beta (PIK3R2), cAMP-dependent protein kinase catalytic subunit alpha (Prkaca) and cAMP-dependent protein kinase type II-alpha regulatory subunit (Prkar2a), were downregulated. Only one canonical pathway was shown to have an increased activity, which is the apelin adipocyte signalling pathways (z-score = 1). Interestingly, both tricarboxylic acid (TCA) cycle (*p* = 1.09E-09) and mitochondrial dysfunction (*p* = 7.31E-09) were the most significantly affected pathways. The latter pathway is represented by numerous proteins expressed in the respiratory chain complex I (NADH dehydrogenase [ubiquinone] 1 alpha subcomplex subunit 8; Ndufa8 and NADH dehydrogenase [ubiquinone] flavoprotein 1, mitochondrial; NDUFV1), complex II (Succinate dehydrogenase [ubiquinone] iron-sulfur subunit, mitochondrial; Sdhb), complex III (Cytochrome b-c1 complex subunit 1, mitochondrial; UQCRC1), complex IV (Cytochrome c oxidase subunit 6A1, mitochondrial; Cox6a1) and ATP synthase complex V (Mitochondrial ATP synthase subunit epsilon and d, Atp5e and Atp5h, respectively).

Further analysis of the biological and disease functions associate the differentially expressed retinal proteins to top ten networks involved in ophthalmic diseases (*p* = 5.69E-09), post-translational modifications (*p* = 6.02E-09), protein folding (*p* = 6.02E-09), RNA damage and repair (*p* = 4.92E-08), protein synthesis (*p* = 4.11E-07), nervous system development and function (*p* = 5.36E-07), visual system development and function (*p* = 5.36E-07), cellular compromise (*p* = 5.09E-06), neurological disease (*p* = 1.41E-05), as well as in inflammatory response (*p* = 2.60E-05) (Fig. 4d).

### 3.4 Comparative Analysis of the Top Canonical Pathways and Diseases and Biological Functions between Ophthalmic Arterial and Retinal Proteome

**Fig. 5:**
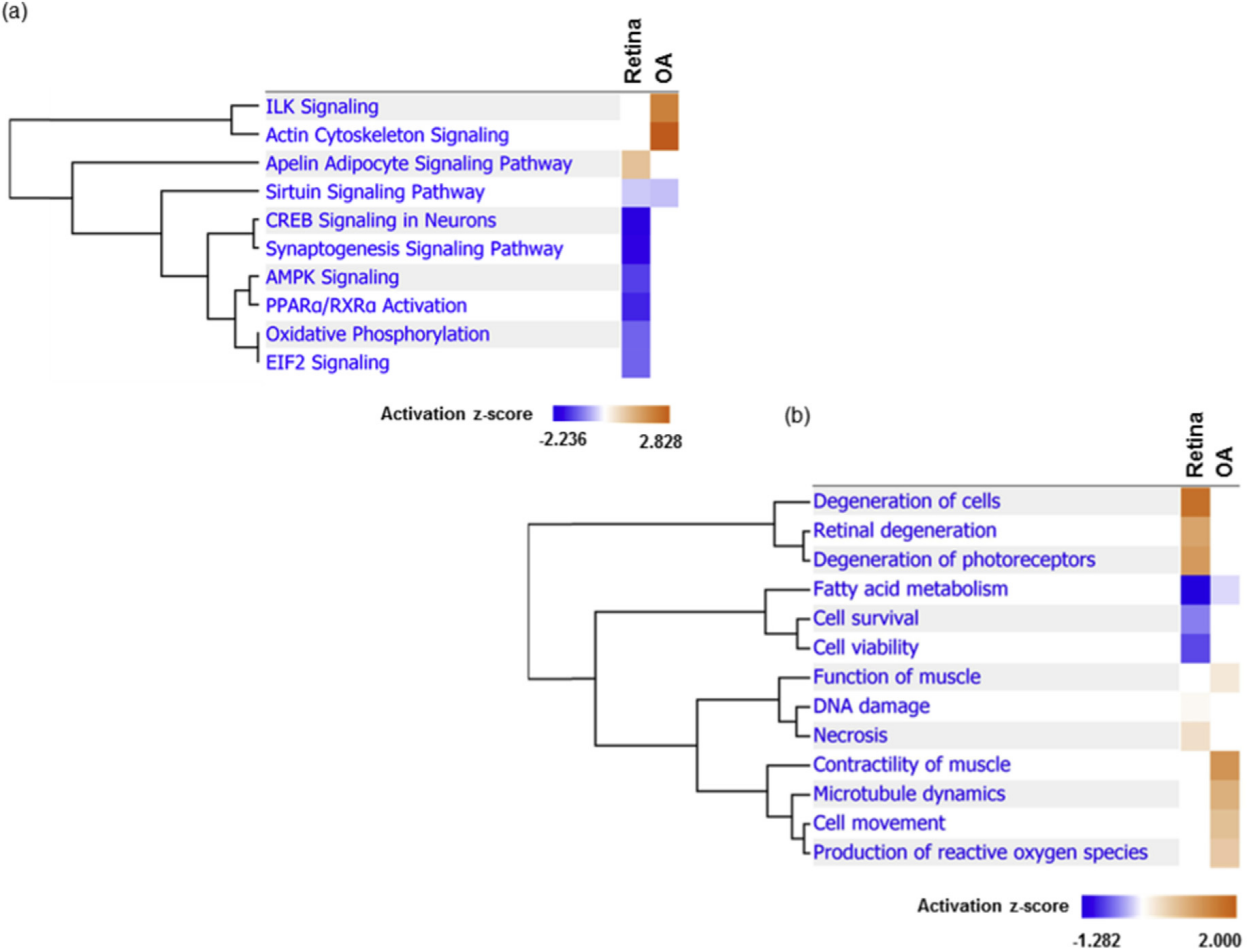
**Comparison analysis**. The hierarchical clustering of the most significantly affected (a) canonical pathways and (b) diseases and biological functions comparing both ophthalmic artery and retina. Blue indicates negative and orange indicates positive regulation based on the activation z-scores of < -2 and > 2 for canonical pathway comparison and < -1 and > 2 for diseases and biological functions. OA: Ophthalmic artery. (For interpretation of the references to colour in this figure legend, the reader is referred to the Web version of this article.)

Next, in an attempt to compare the functionalities of the differentially expressed protein clusters between both ocular vascular beds, the IPA tool was used to analyse the canonical pathways and, diseases and biological functions. Fig. 5a illustrates the top ten significantly implicated canonical pathways in the retina compared to the ophthalmic artery. A striking feature of these results is that, except for the apelin adipocyte signalling pathway (z-score = 1), which was activated, all other pathways were susceptible to be negatively affected by Ang II treatment in the retina, as shown by the inhibition of the following pathways in descending z-score order: cAMP-response element binding protein (CREB) signalling in neurons (z-score = -2), synaptogenesis signalling pathway (z-score = -1.89), PPARα/RXRα activation (z-score = -1.633), AMPK signalling (z-score = -1.414), EIF2 signalling (z-score = -1.134), oxidative phosphorylation (z-score = -1.134). On the contrary, the top two significantly affected canonical pathways comprising the actin cytoskeleton signalling (z-score = 2.828) and integrin-linked kinase (ILK) signalling (z-score = 2.121) were activated in the ophthalmic artery in response to Ang II. However, both vasculature showed a similar expression pattern of the inhibition of sirtuin signalling pathway with z-score = -0.378 and -0.447 for retina and ophthalmic artery, respectively.

Intriguingly, as depicted in Fig. 5b, the most significantly implicated diseases and biological functions of the ophthalmic arterial proteins reflect the activated cluster of networks involved in contractility of muscle (z-score = 1.254), microtubule dynamics (z-score = 0.915), cell movement (z-score = 0.725), production of reactive oxygen species (z-score = 0.618) and function of muscle (z-score = 0.251). In contrast, the differentially expressed retinal proteins reflect the activation of retinal degenerative (z-score = 1.066) processes, in particular degeneration of cells (z-score = 1.718) and photoreceptors (z-score = 1.183), necrosis (z-score = 0.355) and DNA damage (z-score = 0.094). Accordingly, the cell viability (z-score = -0.775) and cell survival (z-score = -0.53) processes were shown to be inhibited. In both vascular beds, the fatty acid metabolism was inhibited, with a significantly greater inhibition in the retina (z-score = -1.282) compared to the ophthalmic artery (z-score = -0.152)

### 3.5 Predicted upstream regulators

**Fig. 6:**
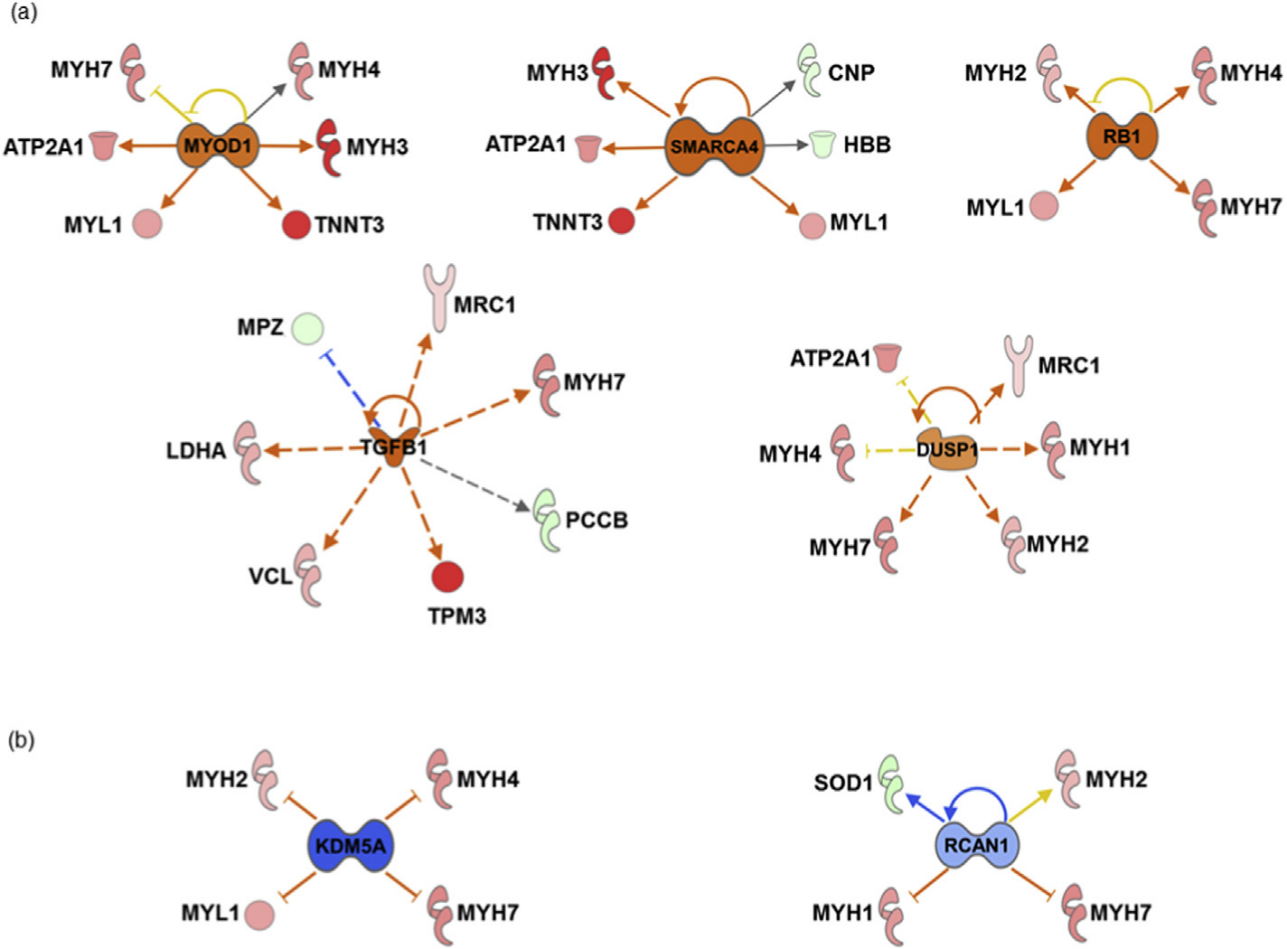

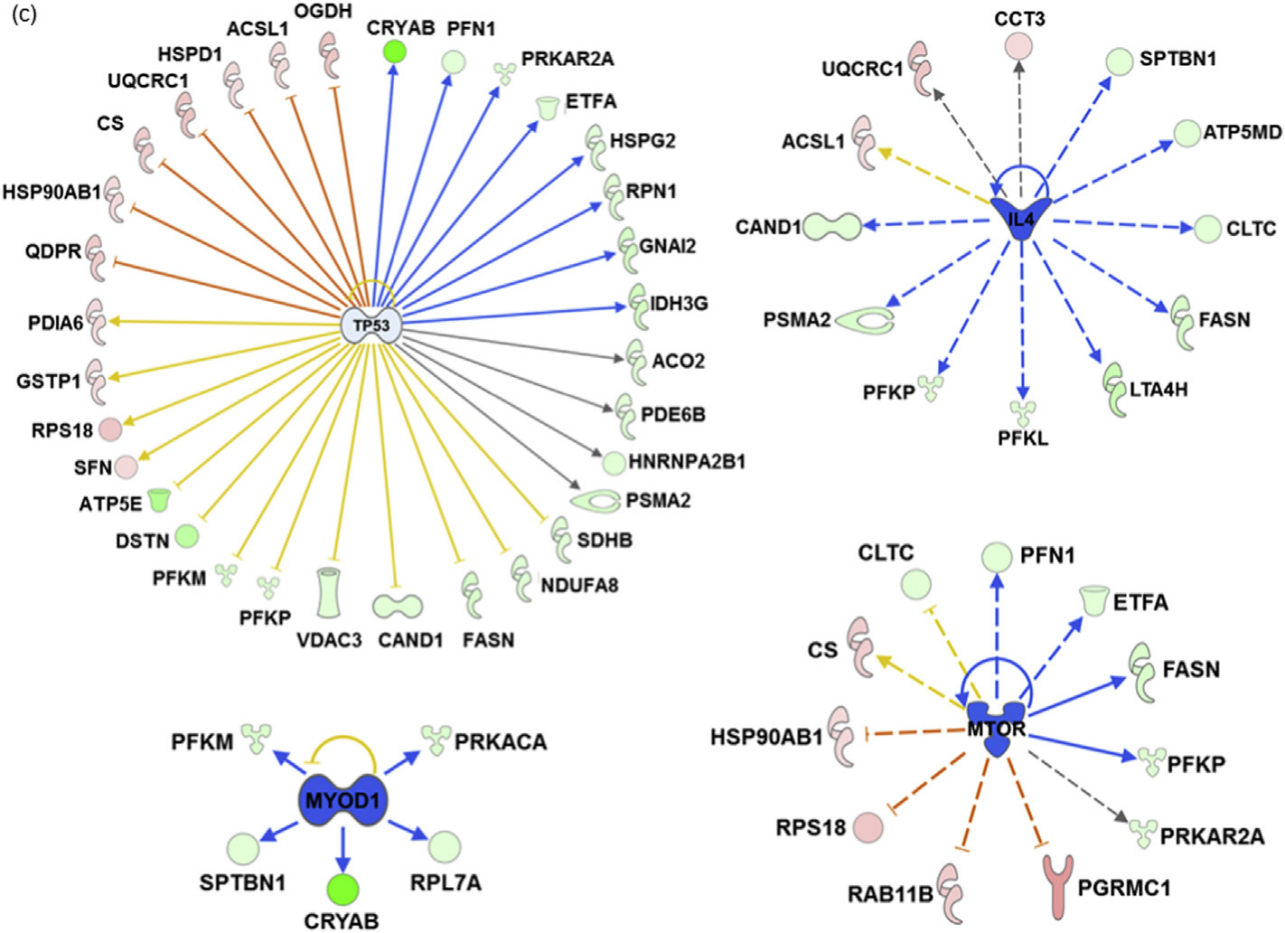

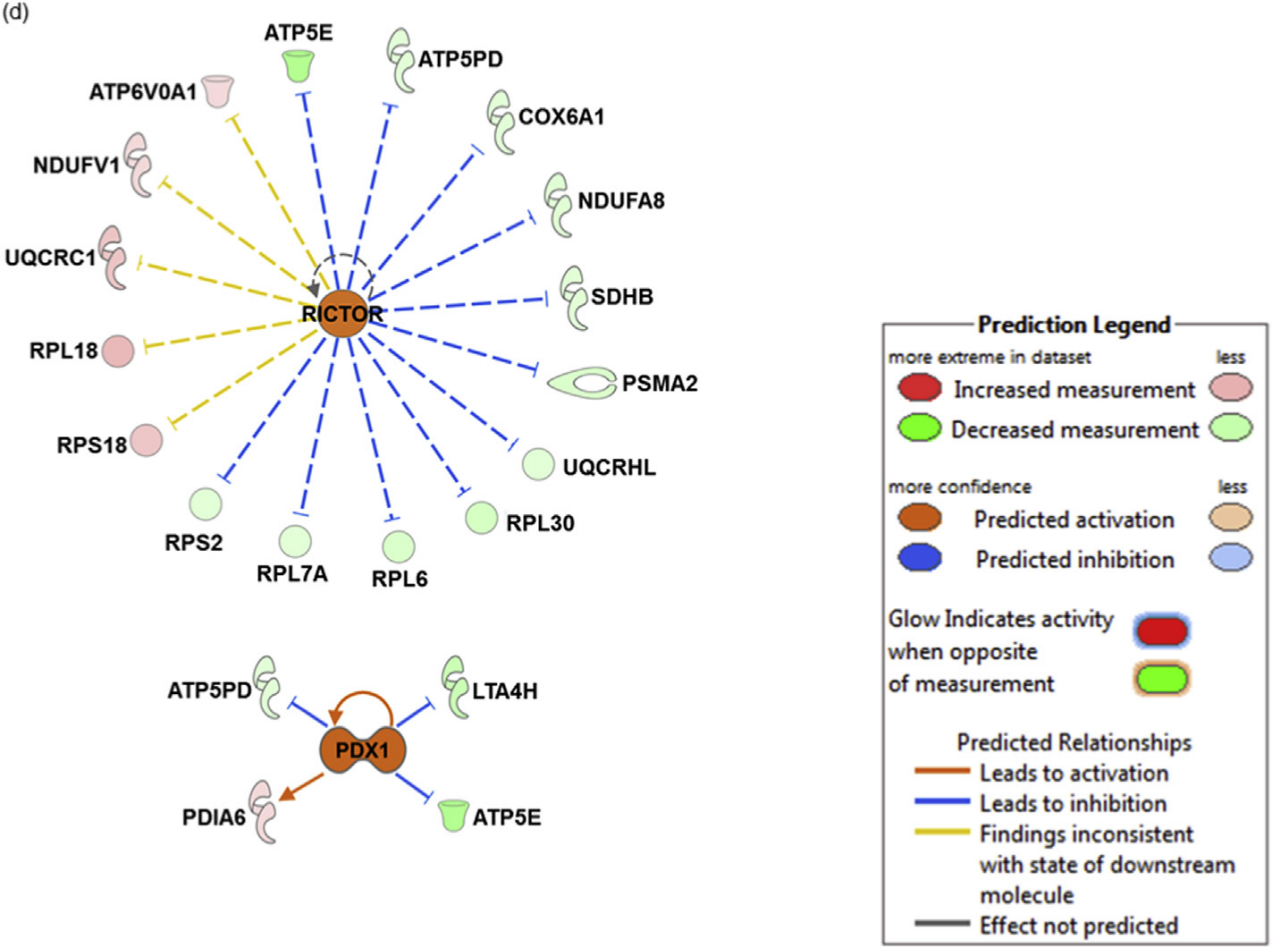
**Predicted upstream regulators**. Interaction networks illustrate the significantly affected predicted upstream regulators for the various clusters of differentially expressed proteins. Top selected upstream regulators predicted to be (a) activated and (b) inhibited in the ophthalmic artery, (c) inhibited and (d) activated in the retina. The colours red and green of the nodes represent the up- and down-regulation of the differentially expressed proteins implicated in the pathways, respectively. The predicted activity of the regulators is shown as orange (activated) and blue (inhibited). (For interpretation of the references to colour in this figure legend, the reader is referred to the Web version of this article.)

Finally, the upstream regulator analysis tool integrated in IPA was instrumental in identifying the most significantly predicted upstream molecules, including transcription factors (TFs), which are likely to govern the regulation of specific clusters of differentially expressed proteins in both ophthalmic artery and retina. In the ophthalmic artery, the large majority of highly implicated upstream regulators comprised transcription regulators. Among these TFs, MYOD1 (*p* = 3.86E-07; z-score = 1.491), SMARCA4 (*p* = 3.17E-04; z-score = 2) and RB1 (*p* = 4.02E-03; z-score = 2) were activated, while RCAN1 (*p* = 6.42E-08; z-score = -0.581) and KDM5A (*p* = 4.37E-05; z-score = -2) were inhibited. Additionally, TGFß1 (a growth factor; *p* = 1.69E-02) and DUSP1 (a phosphatase; *p* = 1.80E-08) were also activated with z-scores 2.399 and 0.984, respectively (Fig. 6a and b). In the retina, four key upstream regulators comprising TP53 (*p* = 8.13E-11; z-score = -0.121), mTOR (*p* = 7.65E-07; z-score = -1.897), IL4 (*p* = 2.22E-03; z-score = -2.538) and MYOD1 (*p* = 2.17E-03; z-score = -2.216) were inhibited (Fig. 6c), while RICTOR (*p* = 1.66E-13; z-score = 1.5) and PDX1 (*p* = 1.25E-02; z-score = 2) were activated (Fig. 6d).

### 3.6 qPCR verification of selected markers

**Fig. 7:**
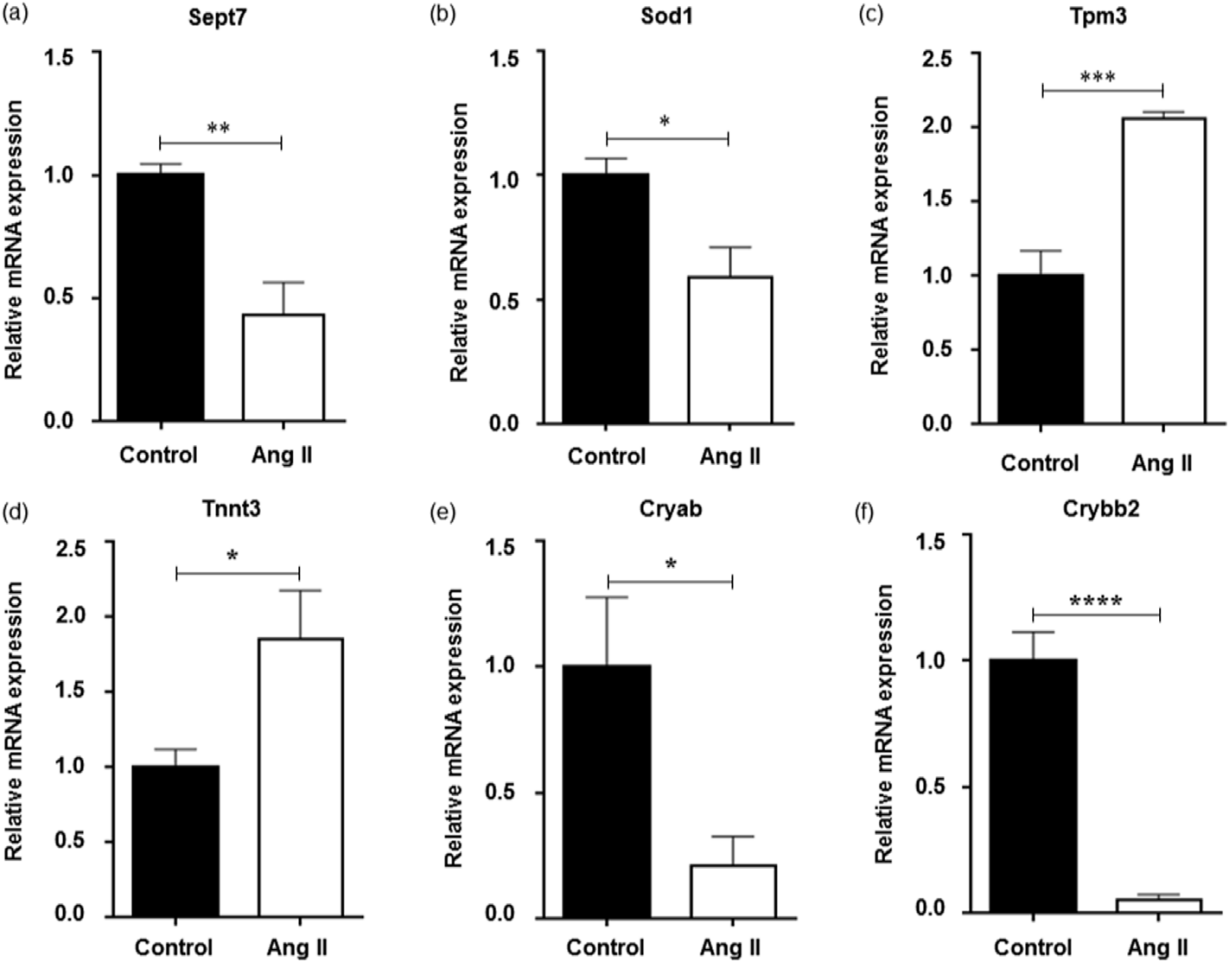
**qPCR validation**. To verify the results of proteomic analysis and to analyze the effects of AngII treatment on mRNA transcription, we performed quantitative real-time PCR analysis of the selected candidates identified to be highly significant and specific in expression in the respective vascular tissues. Bar charts depict the mRNA expressions of the ophthalmic artery comprising (a) *Sept7* (b) *Sod1* (c) *Tpm3* and (d) *Tnnt3*, and retina, which was composed of (e) *Cryab* and (f) *Crybb2*. The values are displayed as mean ± SEM; N=25 (n=5 biological replicates with a pool of 5 samples per replicate). *p < 0.05; **p < 0.01; ***p < 0.001; ****p < 0.0001.

We sought to validate our MS-based proteomicfindings using a second independent method by qPCR analysis of selected candidates, which were identified to be highly significantly differentially expressed and of physiological importance in both vascular beds. The gene expression levels of *Sept7, Sod1, Tnnt3* and *Tpm3* in the ophthalmic artery were consistent with their protein abundance, where the former two genes were verified to be significantly downregulated and the latter two genes were significantly upregulated (Fig. 7a–d). On the other hand, stimulation with Ang II significantly down-regulated the mRNA expression of the *Cryab* and *Crybb2* (Fig. 7e–f) in the retinal samples, which is consistent with the differential expression of these proteins. On the contrary, the expression of *Cryaa* was not affected at the mRNA level (Supplementary Fig. 1). It is noteworthy that the expressions of these selected genes were highly specific to the respective tissue types, as demonstrated by the non-significant expressions of the same genes in the respective counterpart samples (Supplementary Fig. 2).

## 4 Discussion

The octapeptide Ang II derived from the multifunctional RAS is a pleiotropic molecule with the potency to induce a myriad of cellular responses, particularly in the circulatory system [[27], [28], [29]]. Therefore, there have been considerable efforts in the past decades directed towards the study of Ang II-induced oxidative stress and the underlying alterations in the functionality of various blood vessels, which have steadily expanded our understanding of the role of Ang II in different tissues and cells [[30], [31], [32], [33], [34]]. In line of these investigations, the present study endeavoured to elucidate the integrated cellular response to acute Ang II administration on two major ocular vascular beds comprising the ophthalmic artery and retina using a comprehensive proteomics approach. Our data led to two major findings. First, the patterns of retinal proteome changes and differentially expressed proteins provide evidence that acute Ang II treatment culminated in altered tissue bioenergetics and ultimately led to cellular demise. Second, Ang II administration significantly affected proteins implicated in actin cytoskeleton dynamics in the ophthalmic artery. These results come as no surprise as it is well-known that the retina is one of the most energy-demanding tissues and as such, is acutely susceptible to shifts in its energy metabolism [[35], [36], [37]], whereas, as demonstrated by our previous studies, the ophthalmic artery has the inherent capacity to compensate for the lack of its predominant vasodilatory mediators *via* various calcium-mediated signalling mechanisms [38,39]. Nonetheless, this study provides the first glimpse of mechanistic insight into the role and signalling axis of various proteins that opens up new and intriguing avenues for better understanding of the observed changes.

In the retina, a prominent downregulation of proteins from the family of heterogeneous crystallins has been observed, which has also been validated *via* qPCR, particularly Cryab and Crybb2. Although mounting evidence from multiple studies supports a role for stress-induced upregulation of the crystallins as a cell-survival mechanism in the eye [[40], [41], [42], [43]], a tenable supposition for the currently observed decreased levels of these proteins is that Ang II elicited a breach in the retinal cell defence mechanisms and thereby, evoked degeneration. This result is further corroborated by the comparative analysis of the disease and biological functions of the differentially expressed proteins between ophthalmic artery and the retina, which explicitly highlighted the activation of retinal cell degeneration and inhibition of cell survival and metabolic responses. Collectively, this finding is a reminiscent of similar phenomena previously reported in experimental models of retinal degeneration, where alpha-crystallins were found to be significantly downregulated [44,45]. Furthermore, knockout animals lacking the alpha-crystallin genes demonstrated an increased rate of retinal ganglion cell death [46]. Another striking feature in this finding is the cellular localization of the usually cytoplasmic Cryab in the nucleus, which is consistent with its translocation from the cytoplasm to nucleus in stressed conditions [[47], [48], [49]].

The ramifications of Ang II-induced oxidative stress on the retinal proteome were further demonstrated by the dysregulation of numerous energy-producing and metabolic signalling pathways, hinting to a possible shift in bioenergetics. Multiple cellular pathways involved in active energy production comprising the TCA cycle, oxidative phosphorylation, EIF2 signalling, AMPK signalling, the sirtuin signalling and the CREB signalling were affected following Ang II treatment in the present study. Correspondingly, mitochondrial dysfunction was one of the most significantly implicated canonical pathways and previous studies lend credence to the notion that Ang II stimulates the latter in various tissues [50]. Moreover, the disruption of CREB-mediated signalling, which was also found to be inhibited in our analysis, has been associated with compromised mitochondrial efficiency [51]. Therefore, since the mitochondrion is the site of TCA cycle and oxidative phosphorylation, the retrograde signalling cascades stimulated by the perturbation of these machineries could suggest the onset of retinal degeneration [52,53]. Additionally, the AMPK signalling represents the central cellular metabolic sensor and hence, the inhibition of its downstream signalling exacerbates energy stress [54,55]. This energy crisis in the retinal tissue is exemplified by the downregulation of all the differentially expressed proteins clustered in the latter network. It is noteworthy that a large majority of these proteins are kinases, comprising Pfkl, Pfkm, Pfkp, Prkaca, Prkar2a and PIK3R2, which function as key regulators of glycolysis and are associated with amino acid metabolism and protein synthesis [56]. Under metabolic stress conditions, AMPK regulates myriad energy-restoring signalling cascades, including fatty acid oxidation, aimed at maintaining the cellular metabolic balance [55,57,58]. Consistent with this, the concerted regulation of energy homeostasis by fatty acid metabolism was shown to be inhibited in our study, which presumably reflects the breakdown of the cellular energy homeostasis contributed by the fatty acid axis.

It is also necessary to highlight here that a predicted upstream molecule named tumour protein p53 (p53) was identified to be highly significantly involved in the regulation of a cluster of differentially expressed retinal proteins, comprising mainly of proteins in the TCA cycle (Aco2, Cs, Idh3g and Ogdh), glycolysis (Pfkm and Pfkp) and mitochondrial oxidative phosphorylation (Atp5e, Ndufa8, Sdhb and UQCRC1), which is congruent with its initial function in the coordination of mitochondrial respiration and glycolysis [59]. However, this transcription factor is also well-known as the ‘cellular gatekeeper’ owing to its central role in controlling numerous cellular responses to stressors, including regulation of genes responsible for cell death [60]. In retrospect, several studies have documented the alteration of p53 in retinal ganglion cell death following optic nerve crush [[61], [62], [63]]. Similarly, another predicted upstream molecule, mammalian target of rapamycin (mTOR), was also inhibited in the Ang-II treated group, which substantiated the critical role of the differentially expressed retinal proteins regulated by the latter in response to oxidative stress. Activation of this kinase was found to confer protection from cell death under stressful conditions such as degeneration of photoreceptors [64]. Together, the AMPK-p53-mTOR pathway is a fundamental energy-sensing signalling network that regulates cell metabolic status [59]. These proteomics observations reiterate our hypothesis that acute oxidative stress significantly alters the pattern of energy consumption in the retina, leading to energy exhaustion and cellular demise.

Intriguingly, one of the pathways susceptible to be negatively affected by Ang II in both retina and ophthalmic artery was the sirtuin signalling. This is an interesting finding given that the proteins implicated in this pathway vary between these two vasculatures, suggesting alternate compensatory regulation profiles *via* the same pathway. On one hand, sirtuin-mediated signalling cascades are activated as metabolic sensors, which promote acetylation of metabolic proteins from the TCA cycle, subunits of oxidative phosphorylation complexes and fatty acid oxidation enzymes to maintain homeostasis of cellular energetics during cellular stress [[65], [66], [67], [68]], as seen in the retina. Alternatively, sirtuin signalling is also widely known to play an integral role in the maintenance of cellular redox balance and promotes Ang II-induced vascular remodelling, as shown in the mouse aorta [[69], [70], [71]]. This is reflected in the current investigation by the expression level of one of the major antioxidant enzymes, Sod1, involved in the sirtuin signalling in the mouse ophthalmic artery. To clarify whether this decrease in Sod1 protein level was due to downregulation of mRNA, we performed RT- qPCR analysis of this gene and further confirmed a parallel decrease in its mRNA level. The down-regulation of Sod1 enzyme, also well-known as the predominant copper-zinc isoform of Sod in blood vessels [52,72], has been associated with increased oxidative stress in different tissues and importantly, given the inherent antioxidant property of this enzyme, Sod1 deficiency has been shown to augment Ang II-induced vascular dysfunction in microvessels such as small mesenteric arteries [[73], [74], [75]]. Hence, evidence from multiple studies support the current observation that the sirtuin signalling pathway affected by Ang II in both ocular blood vessels in this study is endowed with exquisite functional diversity that is distinctly regulated within each vascular bed.

Another downregulated ophthalmic arterial protein, whose mRNA expression was unequivocally validated *via* qPCR approach, was Sept7. This protein, which belongs to the highly conserved family of small GTPases, is a key component of the membrane-associated cytoskeleton and thus, interacts with actin and microtubules to regulate proper organization and coordination of cellular movement and shape [[76], [77], [78]]. In the renal microvessels, Sept7 was found to be localized in the endothelial cells of the glomerular capillaries [79]. Previous studies have documented that the decline in cardiac contractility in the zebrafish was attributed to the decrement of this protein [80] and one of the septins down-regulated in the aorta of rats with type 2 diabetes was Sept7 [81]. Since septins are known as an important entity in actin-cytoskeleton dynamics owing to their scaffolding function, depletion of SEPT2 for example caused thinning of the actin filaments in the human dermal microvascular endothelial cells, which was reflective of local membrane remodelling processes [76]. A striking feature in our findings is that, consistent with the literature, both actin cytoskeleton and integrin-linked kinase (ILK) signalling pathways were found to be activated in response to Ang II. The ILK-mediated signalling and associated proteins are known to play complex roles in the modulation of microtubule dynamics [82]. In keratinocytes, perturbations in the functionality of ILK-related mechanisms triggered the loss of extracellular matrix deposition and consequent defects in attachment of matrix [83]. It has been elegantly demonstrated by Bendig and co-workers [84] that ILK is an integral component of the cardiac mechanical stretch sensor. In a study by Wakatsuki *et al*. [85], persistent cardiac hypertrophy evoked by several factors, including Ang II, has been shown to lead to decreased contractility due to detrimental deposition of sarcomeres in an attempt to compensate for hemodynamic overload. Here, we identified transforming growth factor beta 1 (TGF-ß1) as the highly activated upstream regulator molecule, which was predicted to orchestrate the downstream regulation of a cluster of structural proteins, namely Mpz, MYH7, Tpm3 and Vcl. Remarkably, this growth factor is known to be activated by Ang II to stimulate matrix deposition *via* increased fibronectin and collagen synthesis in vascular smooth muscle cells [[86], [87], [88]].

It is noteworthy that the large majority of the proteins implicated in both actin and ILK signalling pathways consisted of upregulated expression of myosins. These actin-dependent molecular motors in vascular muscle are the primary determinant of contraction and hence, it is no surprise that myosins are the crucial link between various signal transduction pathways and actin-cytoskeleton [89,90]. Of note, this cluster of myosin proteins functions closely with two other proteins that were found to be significantly upregulated in the ophthalmic arterial proteome as well as mRNA comprising Tnnt3 and Tpm3. Both proteins are the master regulators of muscle contractility and are involved actively in the stabilization and modulation of the functions of actin filaments [91,92]. The upregulation of Tpm3 and Tnnt3 was reported as a result of diabetic muscle infarction and cardiac stress due to increased production of oxidative radicals in myocardial tissue, respectively [93,94]. Moreover, myosins are also highly sensitive to oxidative insult [95] and on the basis of our results here and in previous studies, we speculate that the activation of these signalling pathways and proteins responsible for muscle contractility is a possible adaptive mechanism and concerted effort to preserve the vascular integrity in response to Ang II-mediated oxidative stress. Taken together, the current findings provide compelling support for the ability of the ophthalmic artery to adapt to Ang II-elicited oxidative insult, while recapitulating our previous studies on its inherent capability to compensate for the lack of predominant molecular mediators to preserve vascular functions [38,39].

One of the limitations of the current experimental model is that the *in vitro* approach employed may not be a genuine reflection of the vascular responses *in vivo*. However, this simple yet reproducible exogenous Ang II administration approach was employed to rule out the contribution of arterial pressure changes, to minimize the influence of neurohumoral factors and to allow the direct examination of the intact arterial segments and retinal tissues *per se* [18,[96], [97], [98], [99]]. Moreover, it has been previously shown that Ang II is unable to pass the blood-retina barrier in the eye [97,98]. Importantly, the present results further challenge the parochial view that Ang II-elicited molecular changes are dependent on its pressor effects and thereby, provide direct evidence that Ang II can produce relatively rapid vascular proteome alterations by a local mechanism that is not due to increased arterial pressure. Another study limitation is that only one dose of Ang II ( 0.1 µM) was employed in our present investigation. This was mainly to exclude the confounding effects of tachyphylaxis, which is an inherent issue with the use of cumulative concentrations of Ang II. The effects of Ang II in small vessels tend to be very small and are susceptible to rapid desensitization/tachyphylaxis, as reported previously [[100], [101], [102]]. Tachyphylaxis has seriously impeded the complete assessment of dose-response relationships *in vitro*, which was not seen to repeated infusions of Ang II under *in vivo* conditions [101,102]. Moreover, this Ang II concentration is a standard dosage commonly used in *vitro* experiments [[103], [104], [105]]. Hence, we used 0.1 µM as the optimum, standard dose to elicit a significant response in our samples. Finally, we did not examine the potential reversal of oxidative stress-induced detrimental effects using ROS scavenging agents such as TEMPOL. This is because we wanted to first establish an experimental paradigm that is functional and reproducible for different ocular vascular tissues, and also to ensure that there are significant proteome changes evoked by acute Ang II administration that warrant further investigation. Nevertheless, further studies on the effects of Ang II administration *in vivo* and also the ameliorating influence of oxidant scanvengers will be the focus of our future investigation.

In conclusion, the findings emerging from our study has demonstrated that Ang II-elicited oxidative insult triggers two distinct mechanisms *via* cellular bioenergetics and actin cytoskeleton-mediated vascular remodelling in the retina and ophthalmic artery, respectively. It remains to be determined whether these molecular changes observed in both ocular vascular beds are pathogenic or represent stress-induced pro-survival adaptive responses.

## CRediT authorship contribution statement

**Natarajan Perumal:** Conceptualization, Supervision, Data curation, Formal analysis, Investigation, Methodology, Writing - original draft, Writing - review & editing. **Lars Straßburger:** Investigation, Writing - review & editing. **David P. Herzog:** Formal analysis, Methodology, Investigation, Writing - review & editing. **Marianne B. Müller:** Resources, Writing - review & editing. **Norbert Pfeiffer:** Resources, Writing - review & editing. **Franz H. Grus:** Resources, Writing - review & editing. **Caroline Manicam:** Conceptualization, Project administration, Funding acquisition, Data curation, Supervision, Formal analysis, Investigation, Methodology, Writing - original draft, Writing - review & editing.

## Declaration of competing interest

The authors declare that no competing interests exist.
