# Supplementary material for: Bioenergetic shift and actin cytoskeleton remodelling as acute vascular adaptive mechanisms to angiotensin II in murine retina and ophthalmic artery
Source: Redox Biol. 2020 May 29;34:101597. doi: 10.1016/j.redox.2020.101597 (PMC7327981; doi:10.1016/j.redox.2020.101597)
Supplement: Multimedia component 6 [file mmc6.pdf]

# Supplementary Figures

## Bioenergetic Shift and Actin Cytoskeleton Remodelling as Acute Vascular Adaptive Mechanisms to Angiotensin II in Murine Retina and Ophthalmic Artery

Natarajan Perumal[a], Lars Straßburger[a], David P. Herzog[b], Marianne B. Müller[b], Norbert Pfeiffer[a], Franz H. Grus[a] and Caroline Manicam[a*]

[a]Department of Ophthalmology, University Medical Centre of the Johannes Gutenberg University Mainz, Mainz, Germany.

[b]Department of Psychiatry and Psychotherapy & Focus Program Translational Neurosciences (FTN), University Medical Centre of the Johannes Gutenberg University Mainz, Mainz, Germany.

*__Corresponding author:__

Dr. Caroline Manicam
Department of Ophthalmology,
University Medical Centre of the Johannes Gutenberg University Mainz,
Langenbeckstr. 1,
55131 Mainz, Germany.
E-mail: caroline.manicam@unimedizin-mainz.de
Tel: +49 6131 178279

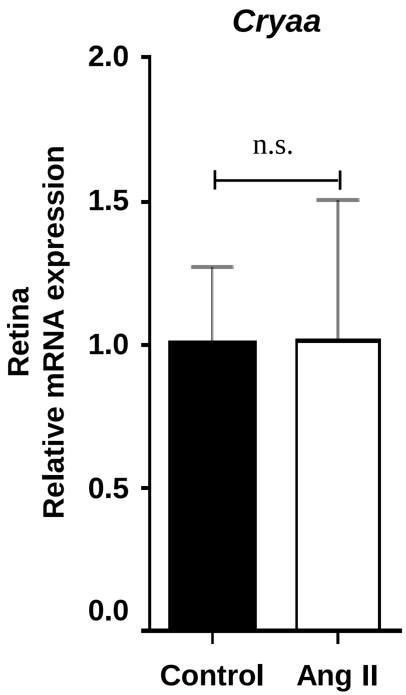


**Supplementary Figure 1 Retinal mRNA expression of *Cryaa***

Bar chart shows the gene expression of *Cryaa* in the retina treated with Ang II compared to vehicle-treated control. n.s. non-significant. The values are displayed as mean ± SEM; N=25 (n=5 biological replicates with a pool of 5 samples per replicate).

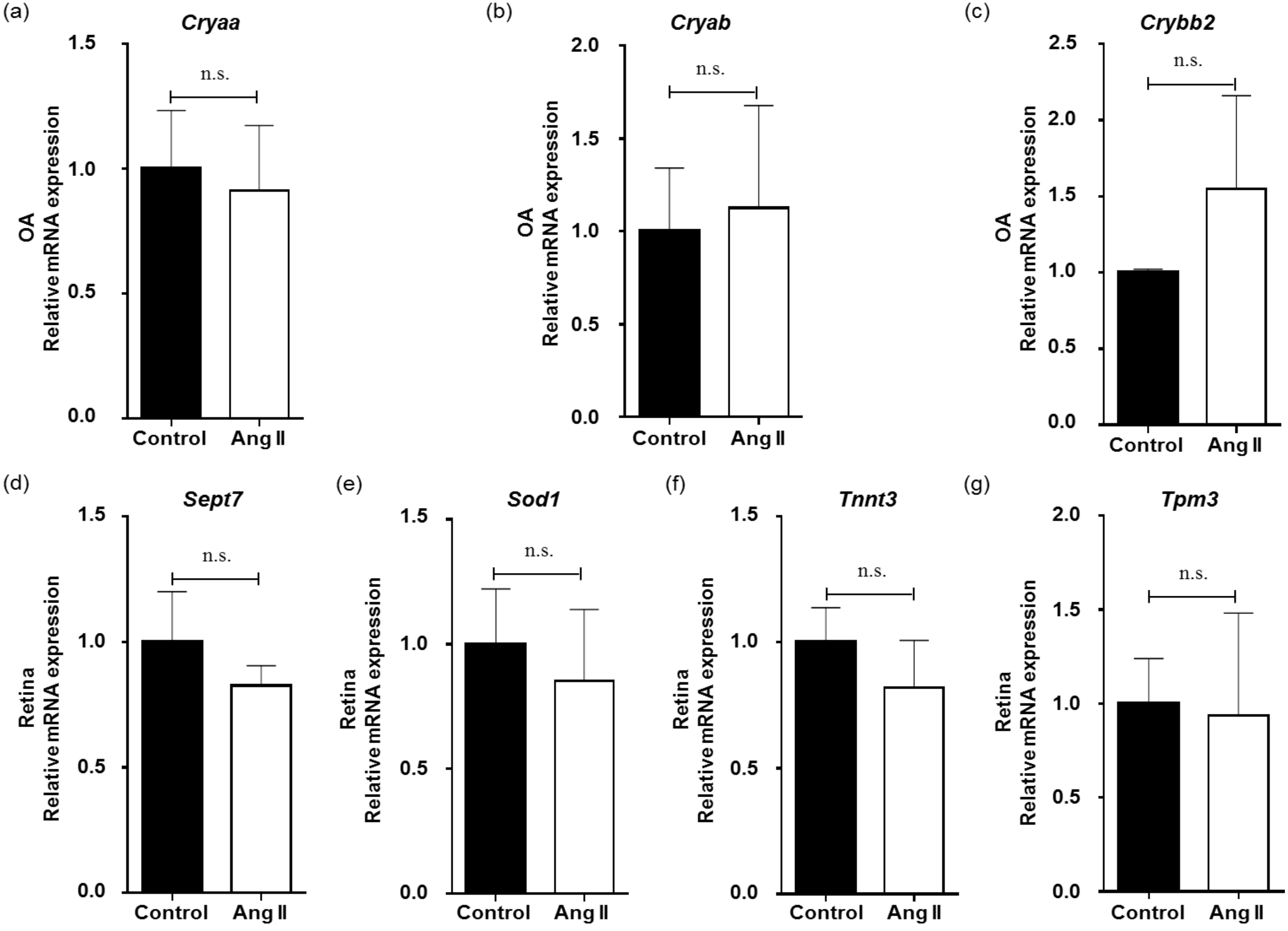


**Supplementary Figure 2 Non-significant mRNA profiles of selected candidates in ophthalmic artery and retina**

Bar charts shows the gene expression of retinal-specific candidates comprising (a) *Cryaa,* (b) *Cryab* and (c) *Crybb2* in the ophthalmic artery, and gene expression of ophthalmic arterial-specific candidates comprising (d) *Sept7,* (e) *Sod1*, (f) *Tnnt3* and (g) *Tpm3* in the retina treated with Ang II compared to vehicle-treated control. n.s. non-significant. The values are mean ± SEM; N=25 (n=5 biological replicates with a pool of 5 samples per replicate).
